# Upper limb ischaemia: a South African single–centre experience

**DOI:** 10.5830/CVJA-2017-049

**Published:** 2018

**Authors:** Tinus du Toit, Kathryn Manning, Nadraj G Naidoo

**Affiliations:** Department of Surgery, Groote Schuur Hospital, Cape Town, South Africa; Department of Medicine, Groote Schuur Hospital, Cape Town, South Africa; Vascular Unit, Department of Surgery, Groote Schuur Hospital, Cape Town, South Africa

**Keywords:** upper limb, acute ischaemia, chronic ischaemia, revascularisation, non-traumatic

## Abstract

**Objective:**

The aims of this study were to report on our experience with upper limb ischaemia (ULI), to define the pattern and distribution of disease, describe key demographic features and report on conventional clinical outcomes.

**Methods:**

This was a single–centre, retrospective, descriptive study. All patients (n = 64) who underwent a surgical intervention for ULI over a 12–year study period were included. Findings were analysed and compared with the current literature.

**Results:**

A male:female ratio of 0.60 was reported. Two major subgroups of patients were identified. The patients in the thrombo–embolic subgroup (n = 30) were notably younger than expected (mean age 55 years) compared to those in the atherosclerotic occlusive disease subgroup (n = 12, mean age 57 years). Presentation overall was generally late, with 8.6% of acute ULI and 48.3% of chronic ULI patients presenting with irreversible ischaemia and tissue loss, respectively. Thrombo–embolism was the dominant vascular pathology reported in this case series (47%). Ninety–five procedures were performed in 64 patients (89 open, six endovascular). Peri–operative (30–day) mortality rate was 7.8%. Systemic and procedure–related complications were observed in 13 and 23%, respectively. The overall major amputation rate was 10.9%. Adherence to follow up was poor (51% at six months).

**Conclusion:**

Although few firm conclusions could be drawn, this review has expanded our overall perspective of ULI, specific to the population we serve. Collaboration between African vascular units should be encouraged in an attempt to further define the pattern of ULI by identifying distinct geographical confounders.

Upper limb ischaemia (ULI) is a relatively uncommon but well recognised vascular clinical entity caused by a wide range of vascular pathologies.^1^ Upper–limb revascularisation procedures comprise approximately 4% of all vascular procedures performed.^2^ Contemporary vascular literature has focused predominantly on vascular occlusive disorders of the lower extremity.

The occupational ramifications and impact on quality of life in those affected can be substantial, often resulting in loss of independence and/or livelihood. A thorough understanding of this condition is essential if significant improvement in surgical outcome and limb functionality is to be made.

Series that combine acute and chronic ischaemia are rare, with most publications reporting on either a single clinical (acute or chronic) or aetiological (traumatic or non–traumatic) aspect of ULI. The majority of publications originate from developed countries, with no reports identified from the African continent to date. Ethnic, demographic and geographic confounders may influence vascular disease development, necessitating further investigation rather than extrapolation. Accordingly, we report on our institutional experience in the context of the current literature and offer a glimpse into several distinctive features specific to the population we serve.

## Methods

Consecutive patients who underwent a surgical intervention for ULI over a 12–year period were identified from the Vascular Unit’s prospectively maintained operative database. Patients presenting with primary Reynaud’s phenomenon and traumatic vascular injuries were excluded. The Trauma Unit at our facility published extensively on this topic within the study period and duplication of data was a concern.^3^–^5^

Qualitative and quantitative data were collected and appropriately coded to assist data analysis using Stata/SE version 13.1 (StataCorpR, College Station, Texas). Frequencies and percentages were calculated for categorical data. Means with minimum and maximum range were calculated for continuous data.

## Results

**Fig. 1:**
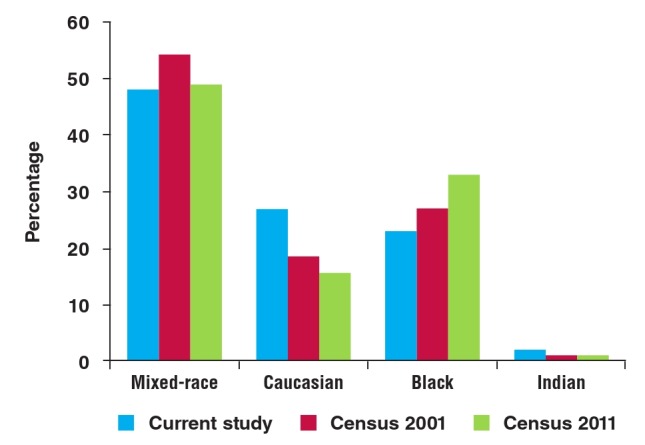
Racial prevalence.

Sixty–four patients presenting with ULI were evaluated and managed surgically from January 2000 to December 2011. Forty females (62.5%) with a mean age of 51 years (range 15–84 years) and 24 males (37.5%) with a mean age of 46 years (range 15–76 years) were included in the study, reflecting a male–to–female ratio of 0.60 (as opposed to 0.96 in the general Western Cape population).^6^ Fig. 1 represents the ethnic distribution within the study group, in comparison with the general population.^6^,^7^

A wide range of vascular pathologies was identified, with noticeable demographic differences between groups (Table 1).

**Table 1:** Comparative demographic details according to vascular pathology

*Pathology*	*Number of Patients*	*Mean age (range)*	*Male:female Ratio*
Thrombo-embolic disease	30	55 (37–80)	0.43
Atherosclerotic disease	12	57 (39–84)	0.71
Thoracic outlet syndrome	8	28 (15–59)	1.67
Takayasu’s disease	4	27 (20–36)	0.33
Thrombo-angiitis obliterans	4	46 (36–53)	3.00
Small-vessel disease	3	32 (31–49)	0.50
Iatrogenic	2	–	1.00
Polymyositis compartment syndrome	1	–	–

A total of 30 patients presented with thrombo–embolic disease, with all but two patients presenting with acute ULI. The majority of embolic occlusions occurred at the level of the brachial artery bifurcation (n = 17). Nine patients presented with more proximal (two subclavian artery, seven axillary artery) and three with more distal occlusions (two radial artery, one ulnar artery). One patient presented with a blue–finger syndrome. A left–sided predominance was noted across all levels of obstruction with a right–to–left–sided ratio of 1:2.

A distinct proximal distribution of atherosclerotic lesions was observed, with the subclavian artery involved in eight, the axillary artery in one and the brachial artery in three patients. As observed in the thrombo–embolic subgroup, clear left–sided predominance was noted with a right–to–left ratio of 1:5. Morphologically, six lesions were described as stenotic and five as occlusions.

Arterial thoracic outlet syndrome: seven of eight patients presented with underlying bony pathology (five cervical ribs, one anomalous first rib and one old clavicle fracture resulting in a pseudo–arthrosis). Six patients presented with chronic and two with acute ULI.

Four patients were diagnosed with Takayasu’s disease. Three patients presented with upper–limb claudication. One of these claudicants suffered an ipsilateral ischaemic cerebrovascular accident prior to presentation. Level of disease ranged from stenosis of the innominate artery with occlusion of its outflow (one patient) to proximal left common carotid artery stenosis with associated left subclavian artery occlusion (two patients). One patient presented with prosthetic graft sepsis complicated by an acute anastomotic bleed following previous aortic arch reconstruction for aneurysmal disease.

Thrombo–angiitis obliterans: four patients with active digital ulceration due to Buerger’s disease were evaluated and surgically managed during the study period. The average smoking history was 36 pack years.

Small–vessel disease: two patients presented with active digital ulceration in combination with Reynaud’s symptoms and one presented with Reynaud’s symptoms alone. The vascular pathologies identified were a vasculitis (Lupus), a hypothenar hammer syndrome and an atherosclerotic small–vessel disease.

## Clinical presentation

Thirty–five patients (54.7%) presented with acute ULI necessitating surgical intervention. Three patients (8.6%) had signs of irreversible ischaemia (Rutherford grade III ULI) and a further nine (25.7%) were diagnosed with Rutherford grade IIb ULI.

Twenty–nine patients presented with chronic ULI. Fourteen patients (48.3%) presented with tissue loss. Other clinical presentations in this group included claudication (31%), rest pain (13.8%) as well as neurovascular symptoms (6.9%).

## Surgical interventions

Ninety–five procedures were performed in 64 patients. Of these, 89 were open procedures with six cases managed by means of an exclusively endovascular approach. A distinction should be made between minor procedures (10 in total; wound debridements, evacuation of haematomas, closure of fasciotomy wounds and excision/ligation of bypass grafts), attempts at revascularisation (77 in total; see Table 2) and ablative procedures (18 in total; see Table 3).

**Table 2:** Procedures performed according to aetiology (excluding minor and ablative procedures)

*Procedures performed*	*No.*
Thrombo-embolic disease	35
Thrombo-embolectomy (fasciotomy in eight)	25
Brachial–brachial/–distal bypass (autologous vein graft)	4
Catheter-directed/intra-operative thrombolysis	3
Stent–graft placement	1
Aortic arch reconstruction (Gelsoft® Dacron)	1
Veinpatch angioplasty of veingraft	1
Atherosclerotic occlusive disease	19
Subclavian artery stent placement	5
Brachial–distal/brachial–brachial bypass graft	4
Subclavian–axillary/subclavian–brachial bypass graft	3
Axillary–brachial bypass graft	2
Common carotid–axillary/common carotid–brachial bypass graft	2
Graft thrombectomy	2
Subclavian artery balloon angioplasty	1
ATOS (each row represents a patient)	11
Thrombo-embolectomy + fasciotomy; Ipsilateral TO decompression; contralateral TO decompression	3
Thrombo-embolectomy + fasciotomy; Ipsilateral TO decompression	2
Common carotid–brachial RSBG bypass	1
Subclavian–axillary PTFE bypass + brachial–ulnar RSVG bypass	1
Subclavian–axillary PTFE bypass	1
Ipsilateral TO decompression	1
Common carotid–brachial Dacron® bypass + brachial–ulnar RSVG bypass + fasciotomy	1
Takayasu’s disease	4
Aortic arch reconstruction AlboGraft®	2
Redo aortic arch reconstruction SilverGraft®	1
Axillary–axillary PTFE bypass	1
Thrombo-angiitis obliterans	2
Thoracoscopic sympathectomy	2
Small-vessel disease	3
Thoracoscopic sympathectomy	2
Brachial–distal RSVG bypass	1
Iatrogenic (each row represents a patient)	2
Fasciotomy	1
Thrombo-embolectomy + fasciotomy	1
Polymyositis compartment syndrome	1
Fasciotomy	1

TO: thoracic outlet; RBVG: reverse basilic vein graft; RSVG: reverse saphenous vein graft; PTFE: polytetrafluoroethylene.

Surgical outcome was reported by quantifying the mortality, morbidity and amputation rates, and functional outcome at certain time intervals post–initial procedure. Unfortunately, follow–up appointments were poorly attended, restricting the interpretation of long–term data (Table 3).

**Table 3:** Summary of 30-day, six-month and long-term outcome

*Outcome measure*	*30-day n (%)*	*6-month n (%)*	*Long-term n (%)*
Adherence to follow up	53 (83.0)	30 (50.8)	17 (28.8)
Mortality	5 (7.8)	–	1
Acute coronary syndrome	2		
Acute kidney injury	2
Acute respiratory failure	1
Morbidity
Systemic complications	8 (12.5)	–	–
Acute kidney injury	3
Acute respiratory insufficiency	3
Acute coronary syndrome	1
Cerebrovascular incident	1
Procedural complications	18 (23.4)	7	1
Surgical site haematoma	6
Superficial surgical site infection	4
Bypass graft occlusion	3	5	1
Pseudo-aneurysm post-angiogram	2
Delayed fasciotomy	1
Neuropraxia	1	1
Re-thrombosis of native vessels	1	1
Amputation rate
Primary amputation (2 major, 8 minor)	10 (15.6)
Secondary amputation (4 major, 4 minor)	8 (12.5)	1
Functional outcome
Normal		23	13
Contracture		4	2
Claudication symptoms		2	1
Motor weakness		1	1

In the first 30 days, 18 amputations were performed in 64 patients. Ten amputations were performed primarily (at initial surgical procedure) in patients presenting with irreversible tissue necrosis. Eight secondary amputations (four major, four minor) were performed within 30 days following an initial attempt at revascularisation. A total of six patients (9.4%) required a major amputation at 30 days, of whom three presented with acute ULI.

At six month’s follow up, five patients presented with bypass graft occlusion (resulting in one above–elbow amputation) and one with re–occlusion of native vessels post–embolectomy. Functionally, four patients presented with contractures, one with motor weakness (affecting activities of daily living) and two with claudication. Twenty–three patients were assessed as having a fully functional ipsilateral upper limb.

After six months, one patient developed bypass graft occlusion as part of an agonal event. Thirteen patients reported normal function, two presented with contractures, one with persistent motor weakness and one with claudication symptoms.

Five patients died within 30 days of admission, resulting in a 30–day all–cause mortality rate of 7.8%. No further mortalities or systemic complications were noted at the six–month follow up. One patient died of lung carcinoma two years after initial presentation with ULI.

## Discussion

We report on the first institutional experience with surgical management of ULI from the African continent, in an attempt to identify ethnic, demographic and geographic confounders. However, several research limitations resulted in the generation of multiple, tentative assumptions to direct future research, rather than robust scientific conclusions.

Firstly, by attempting to discuss distinctly different aetiopathological processes in unison, important individual characteristics may be obscured. Adherence to follow up was poor, limiting the interpretational value of long–term data. With this in mind, a few relevant findings will be discussed.

The true incidence of ULI in South Africa remains speculative. A major limiting factor is the paucity of data on non–surgical management of ULI. In the current series, subjects were retrospectively selected from a surgical database without capturing those managed non–surgically. The singlecentre nature of this series does not allow for any firm conclusion regarding the regional and race–specific incidence of ULI as the number and ethnicity of patients seeking medical attention from private healthcare facilities are currently unknown.

Despite the above–mentioned limitations, a clear escalating trend was observed, with 56% of surgically managed patients referred within the last four years of the study period. An increase in the absolute number of referrals is the most conceivable explanation for the observed trend. A less likely explanation may be that a more aggressive surgical approach was followed during the last four years of the study. Anecdotally though, the indications for surgical intervention have remained unchanged.

The largest surgical series investigating patients undergoing revascularisation procedures for ULI was published by Deguara et al.^1^ in 2005. A total of 172 patients were included over a 20–year period, with 53 cases related to upper–extremity trauma (excluded in the current series). Comparison of data between the two series makes for interesting discussion, especially when thrombo–embolic and atherosclerotic occlusive disease subgroups are analysed (Table 4).

**Table 4:** Demographic and outcome comparisons of thrombo-embolic and atherosclerotic occlusive disease

	*Number*	*Mean age (years)*	*M:F Ratio*	*30-day mortality rate (%)*	*Amputation rate (%)*
Current series
Thrombo-embolic	30	55	1:2.3	16.7	13.3
Atherosclerotic occlusive	12	57	1:1.4	0	8.3
Deguara et al.^1^
Thrombo-embolic	61	72	1:1.1	18.2	0
Atherosclerotic occlusive	29	63	1:1.9	6.9	0

Multiple publications support the view that patients with thrombo–embolic ULI, when compared to athrosclerotic occlusive disease, present at a more advanced age.^1^,^8^ Interestingly, this finding was not observed in our series. Possible explanations include the assumed impact that a relatively low life expectancy (57.7 years for males and 61.4 years for females)^6^ may have, as well as the suspicion of a different risk–factor profile compared to other research populations.

Both tuberculosis^9^ and HIV infection^10^ have been identified as acquired hypercoagulable states. Therefore, with the prevalence of tuberculosis (25/1000)^11^,^12^ and antenatal HIV infection (33%)^13^ in the Western Cape on the rise,^14^ it is conceivable that the study population is at higher risk of developing thrombo–embolic disease. In the absence of a national registry, a prospective survey specifically designed to evaluate the impact of tuberculosis and HIV/AIDS on the incidence and pathogenesis of ULI should be performed.

Furthermore, all five of the 30–day mortalities were observed in the embolic acute ULI subgroup. The concept that mortality following embolectomy is a consequence of the patient’s co–morbidity rather than the embolus itself, is well supported.^1^,^15^ In our series, post–embolectomy mortality was attributable to acute coronary syndrome (n = 2), acute kidney injury (n = 2) and acute respiratory failure (n = 1), resulting in a 30–day all–cause mortality rate of 16.7%. These findings are in keeping with recent international literature, ranging between eight and 19%.^1^,^16^,^17^ The only death observed in the chronic ULI group was as a result of lung carcinoma, documented two years after initial surgery for atherosclerotic occlusive disease.

Ablative procedures were reported as either primary (performed at initial procedure) or secondary (following an attempt at revascularisation), with digital (minor) and aboveor below–elbow (major) amputations separately recorded. The 30–day amputation rate following an attempt at revascularisation was 12.5%, with major (both primary and secondary) amputations performed in 6.3%.

Patients generally presented late, with 8.6% in the acute ULI group and 48.3% in the chronic ULI group presenting with irreversible ischaemia and tissue loss, respectively. When comparing surgical outcome to that of other case series (see Fig. 2), one has to consider indications for surgery. Units implementing a more aggressive approach to relatively minor symptoms may reflect better surgical outcomes, particularly superior limb–salvage rates. No limbs were amputated in the Deguara1 series, but the indications for intervention were not reported.

**Figure:**
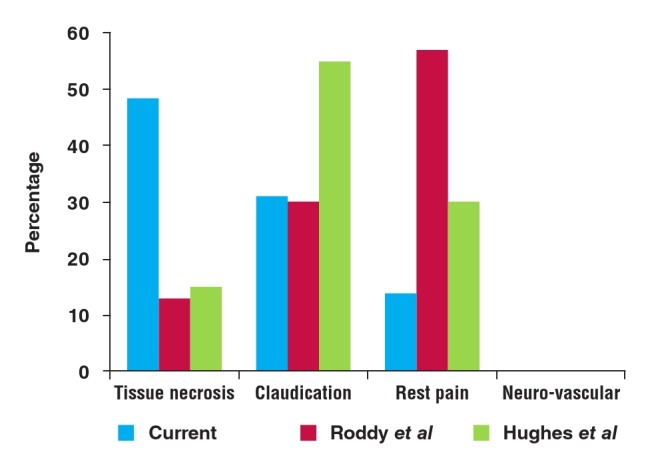
Summary of chronic ULI presentations.

Of the 64 patients included in this review, seven were confirmed to be HIV positive by HIV Ag/Ab Combo (ELISA) testing. However, only 30 patients underwent testing (as indicated by folder laboratory results sheet or NHLS Disa electronic results system). One patient developed superficial surgical site infection and another died of prosthetic graft sepsis, complicated by an acute bleed. Due to the low rate of HIV testing and small number of patients involved, it is not possible to reach firm conclusions regarding clinical outcome in this subgroup of patients.

Candidates for exclusive endovascular management were conservatively selected. Five subclavian artery lesions were managed by primary stent placement, with one lesion stented after failed percutaneous balloon angioplasty. One patient sustained a procedure–related complication in the form of an ipsilateral cerebrovascular incident. All of these patients attended the six–month follow–up appointment and reported normal function of the affected upper limb.

## Conclusion

Although few firm conclusions could be drawn, this review has expanded our overall perspective of ULI, specific to the population we serve. Collaboration between African vascular units should be encouraged in an attempt to further define the pattern of ULI by identifying distinct geographical confounders.
